# Evaluating AI-Generated Patient Education Guides: A Comparative Study of ChatGPT and Deepseek

**DOI:** 10.7759/cureus.85277

**Published:** 2025-06-03

**Authors:** Jaziya Jabeen, Jyothis G Saji

**Affiliations:** 1 Cardiology, Royal Cornwall Hospital, Cornwall, GBR; 2 Emergency Medicine, Jubilee Mission Medical College & Research Institute, Thrissur, IND

**Keywords:** artificial intelligence in medicine, chatgpt, ckd, copd, deepseek, epilepsy, heart failure, patient education guides

## Abstract

Introduction

Artificial intelligence (AI) chatbots, including ChatGPT and DeepSeek, are becoming popular tools for generating patient education materials for chronic diseases. AI chatbots are useful as supplements to traditional counseling but lack the empathy and intuition of healthcare professionals, making them most effective when used alongside human therapists. The objective of the study is to compare ChatGPT-4o and DeepSeek V3-generated patient educational guides for epilepsy, heart failure, chronic obstructive pulmonary disease (COPD), and chronic kidney disease (CKD).

Methodology

In this cross-sectional study, the standardized prompts for each disease were entered into ChatGPT and DeepSeek. The resultant texts were evaluated for readability, originality, quality, and suitability. Unpaired t-tests were performed to analyze statistical differences between tools.

Results

Both AI tools created patient education materials that had similar word and sentence counts, readability scores, reliability, and suitability in all areas, except for the similarity percentage, which was much higher in ChatGPT outputs (p=0.049). The readability scores indicated that both tools produced content that was above the recommended level for patient materials. Both tools resulted in high similarity indices that exceeded accepted academic thresholds. Reliability scores were moderate, and while understandability was high, actionability scores were suboptimal for both models.

Conclusion

The patient education materials provided by ChatGPT and DeepSeek are similar in nature, but neither satisfies recommended standards for readability, originality, or actionability. Both still need additional fine-tuning and human oversight to enhance accessibility, reliability, and practical utility in clinical settings.

## Introduction

Epilepsy, chronic kidney disease (CKD), chronic obstructive pulmonary disease (COPD), and heart failure are chronic illnesses that affect millions worldwide, necessitating careful monitoring and education. Chronic kidney disease (CKD) and COPD, both progressive conditions, contribute to considerable morbidity and mortality, requiring patients to manage symptoms and follow treatment regimens closely. Heart failure, a leading cause of hospitalization, demands comprehensive patient understanding to optimize care and prevent exacerbations. Patient education is an integral part of managing these conditions, as it helps in the timely recognition of complications, adherence to medication, and renders necessary lifestyle changes, thereby reducing morbidity and mortality. Effective patient education empowers individuals to manage their conditions, improving overall outcomes [1-5].

Artificial intelligence (AI) tools are capable of responding to patient questions through immediate, customized, and evidence-based data, enhancing patient engagement and treatment compliance. They have the ability to decrease healthcare workers’ workload, particularly for roles such as nurses, pharmacists, general practitioners, and medical assistants, by responding to frequent queries. On the other hand, AI is non-empathetic, can provide incorrect or misleading information, particularly when the data it is trained on is outdated or inaccurate, and may struggle with understanding intricate or complicated questions. Moreover, trust in AI among patients can be restricted, and privacy issues regarding sensitive information exist [6]. ChatGPT is a language model based on AI created by OpenAI using machine learning methods to comprehend and produce human text, helping to respond to queries, make recommendations, and hold conversations [7].DeepSeek is an open-source generative AI technology that runs on large language models. It offers continuous learning, offline deployment for improved data privacy, and customization for healthcare use. However, it also raises concerns regarding data security, accuracy, and regulatory compliance [8].

AI chatbots are becoming increasingly important in patient counseling, offering instant, personalized assistance and advice. They assist with daily tasks such as sending medication reminders and supplying basic health information, easing the workload of medical professionals. However, they lack the empathy and human intuition that healthcare professionals provide, making them most effective when used in conjunction with human therapists. Overall, AI chatbots enhance patient counseling by providing cost-effective, non-judgmental support and facilitating more efficient healthcare delivery [9,10].

Many studies have assessed patient education guides generated by ChatGPT [11,12]. In contrast to ChatGPT, the features of DeepSeek have not been extensively explored in the context of patient education guides. This study seeks to fill this gap by comparing the patient education guides generated by ChatGPT and DeepSeek in terms of readability, quality, similarity, and suitability, and by shedding light on the strengths and weaknesses of both AI tools in the field of patient education.

The objective of this study is to compare and evaluate the readability, quality, and similarity of responses provided by ChatGPT and DeepSeek in patient education guides regarding epilepsy, heart failure, COPD, and CKD.

## Materials and methods

Study design

This is an original cross-sectional study designed to evaluate the readability, quality, and similarity of AI-generated responses to patient education guides related to epilepsy, heart failure, COPD, and CKD. There were no human participants in the one-week research (April 10-17, 2025), since the data was entirely derived from two different AI tools: ChatGPT (GPT-4o) and DeepSeek (DeepSeek-V3). Ethics approval was not required as the study did not involve human participants or sensitive data. Data collection and analysis were conducted online, utilizing digital tools and software.

Data collection

The prompt was presented to two free version of AI programs, ChatGPT (version 4.o) [13] and DeepSeek (DeepSeek-V3) [14], on a single day using the Chrome browser with the cache cleared on an ASUS ROG Strix G G731GU laptop connected to stable Wi-Fi (100-150 Mbps). This consistent setup ensured a fair comparison of their ability to generate patient education guides. Each prompt was entered separately into a freshly created AI chatbot account, guaranteeing that no prior responses were present in the discussion history. Each prompt was directed into a separate “chat”. Both the AI tools were asked to produce patient education guides for the four diseases in their default and standard settings, without any fine-tuning or modifications, using the same prompts to ensure consistency in the inputs: “Write a patient education guide for epilepsy”, “Write a patient education guide for heart failure”, “Write a patient education guide for chronic obstructive pulmonary”, and “Write a patient education guide for chronic kidney disease”. The generated responses were recorded in Microsoft Word documents. The outputs were subsequently assessed with standardized tools, including the Flesch Reading Ease Score (FRES) and the Flesch-Kincaid Grade Level (FKGL) for readability [15], Turnitin text-matching software for evaluating content similarity [16], the DISCERN Instrument for quality assessment and PEMAT (Patient Education Materials Assessment Tool) for suitability [17].

Readability assessment

The readability of AI’s response was evaluated using recognized metrics such as the FRES and FKGL. The FRES [18] (from 0 to 100, with higher scores reflecting easier readability) and the Flesch-Kincaid Grade Level [19] (a modified version of the FRES, which measures the years of education needed in American grade school to understand a text) are readability metrics that are applied in the healthcare sector to maximize patient understanding of medical information. These algorithms have been validated in the literature and are used to evaluate the readability of patient-centered medical texts in healthcare [20]. The readability scores were determined by pasting AI's responses into a free, open-access online readability calculator tool (Flesch-Kincaid calculator) [15].

Evaluation of similarity

We turned to investigate the brochures for plagiarism, or the act of copying the work of another person, and the amount. Turnitin’s text-matching software [16] was provided with separate Word documents comprising individual guides for each of the four diseases generated by the two AI tools. Turnitin generated a similarity report that included its overall similarity index (OSI), which is the percentage of text without quote marks and references that matched. The goal was to evaluate whether the AI tools were indeed generating original content or were merely repackaging something that had already been published.

Evaluation of quality

The DISCERN score, an instrument created to grade the quality and reliability of the health-related information available on the internet, was used to determine the quality of AI-generated responses. Its assessment includes 16 questions covering the reliability, completeness, and overall quality of the content, each rated on a 1-5 scale. It helps to assess whether online health resources are trustworthy and comprehensive for the healthcare providers, researchers, and the general public [17]. Two observers assessed the DISCERN score independently with good agreement (Cohen’s Kappa=0.738, p<0.001), and disagreements were settled by consensus decision before the final analysis.

Suitability assessment

PEMAT analysis was conducted to evaluate the appropriateness of the patient education guide. The PEMAT consists of two domains: understandability and actionability. The observer rates topic-wise statements in each domain with a 1 if they agree or a 0 if they disagree. There are very few items that have the option of “not applicable” (NA). The weighted scores are summed to give an understandability and actionability score, ranging from 0 to 100% [21]. The PEMAT was assessed by two independent observers with a near-perfect agreement (Cohen’s Kappa=0.914, p<0.001), and any disagreements were resolved by consensus decision prior to the final analysis. Scores were evaluated by calculating the average of the scores given by the two observers.

Statistical analysis

The collected data will be imported into Microsoft Excel and analyzed using SPSS version 27. The inter-observer agreement for the DISCERN and PEMAT scores was assessed using Cohen’s Kappa coefficient. An unpaired T-test will assess AI-generated responses from the two tools, with a p-value < 0.05 considered statistically significant.

## Results

The characteristics of the responses generated by DeepSeek and ChatGPT are displayed in Table 1. In all the domains examined, including word count, average syllables per word, sentence count, average words per sentence, grade level, ease score, similarity percentage, DISCERN score, understandability, and actionability, only similarity had statistically significant differences.

**Table 1:** Characteristics of the responses produced by ChatGPT and DeepSeek. *P-values <0.05 are considered statistically significant.

Parameters	ChatGPT		DeepSeek		P-value*
	Mean	Standard Deviation	Mean	Standard Deviation	
Words	500.25	72.71	425.25	31.66	0.132
Sentences	68.75	8.62	75.75	9.74	0.323
Average Words per Sentence	7.43	1.58	5.65	0.38	0.117
Average Syllables per Word	1.95	0.06	1.9	0.082	0.363
Grade Level	10.33	0.53	9.03	0.87	0.051
Ease Score	34.35	3.93	40.38	6.66	0.18
Similarity %	46	8.98	32.5	5.26	0.049
DISCERN	47.63	5.11	46.75	5.87	0.829
Understandability %	81.81	6.43	80.15	9.82	0.789
Actionability %	50	0	65.63	18.75	0.194

**Figure 1:**
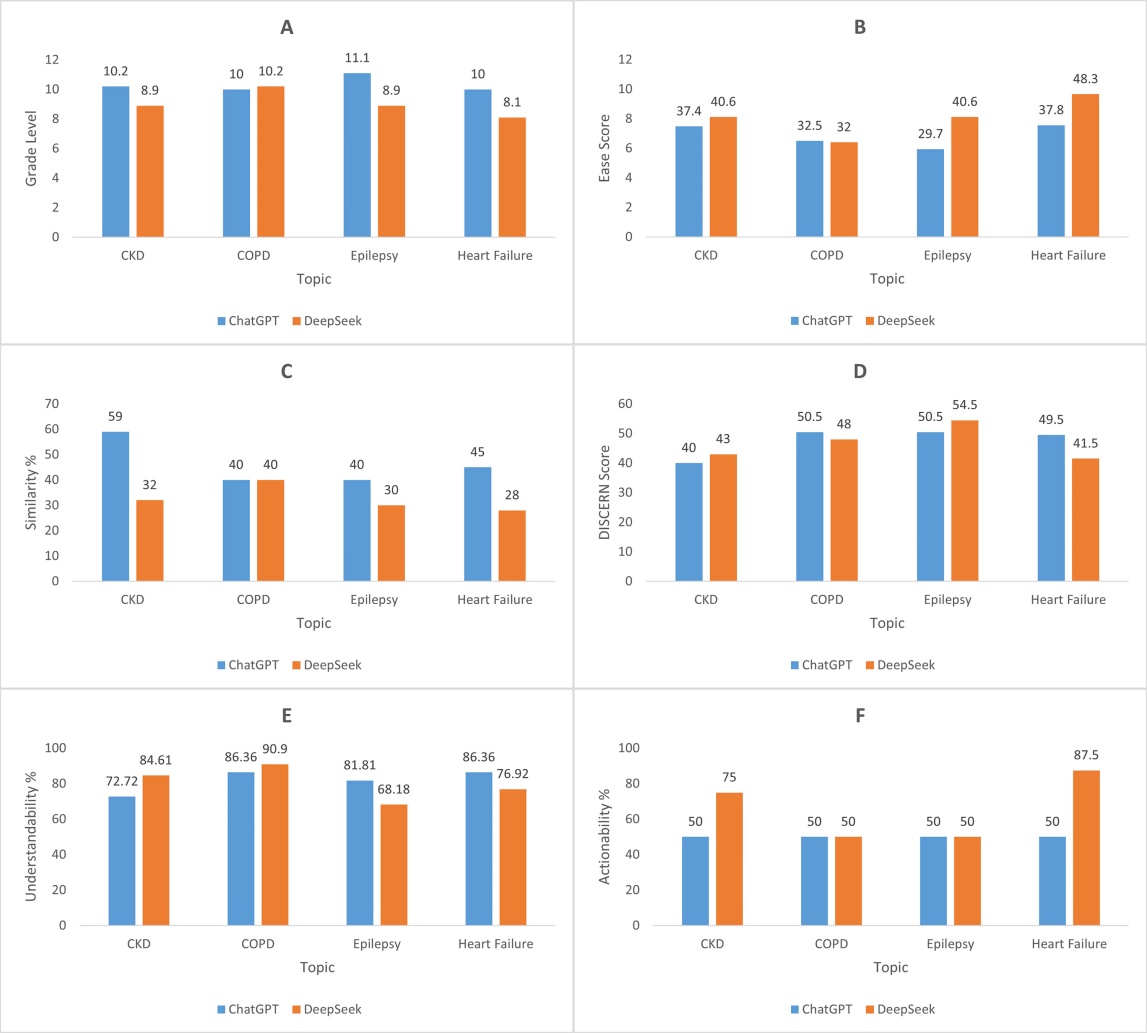
Comparison of the grade level (A), ease score (B), similarity percentage (C), DISCERN score (D), understandability percentage (E), and actionability percentage (F) for the patient education guides produced by ChatGPT and DeepSeek.

A graphical comparison of the grade level, ease score, similarity percentage, DISCERN score, understandability, and actionability for the patient education guides created by DeepSeek and ChatGPT is presented in Figure 1.

Based on the grade level comparison, the results of DeepSeek and ChatGPT were almost identical (Figure 1A). Out of the four diseases, ChatGPT had a higher grade level in three diseases (CKD: 10.2, epilepsy: 11.1, and heart failure: 10), whereas DeepSeek had a higher grade level in one disease (COPD: 10.2).

While assessing the ease score (Figure 1B), DeepSeek’s guide scored slightly greater in three diseases (CKD, epilepsy, and heart failure) than ChatGPT's, with a pronounced difference for epilepsy (ease score of DeepSeek = 40.6, ease score of ChatGPT = 29.7), while the ease score for ChatGPT was slightly greater in COPD (ease score of ChatGPT = 32.5, ease score of DeepSeek = 32). This difference suggested that the text produced by DeepSeek was simpler to read.

When the similarity percentages of the educational guide were compared (Figure 1C), it was observed that ChatGPT exhibited a greater similarity percentage on CKD (59%), epilepsy (40%) and heart failure (45%), whereas both ChatGPT and DeepSeek had the same percentage of similarity on COPD (40%).

While assessing the DISCERN score (Figure 1D), DeepSeek’s guide scored slightly greater in CKD (DeepSeek=43, ChatGPT=40) and epilepsy (DeepSeek=54.5, ChatGPT=50.5) than ChatGPT's, while the DISCERN score for ChatGPT was slightly greater in COPD (ChatGPT=50.5, DeepSeek=48). and heart failure (ChatGPT=49.5, DeepSeek=41.5).

Based on the understandability percentage comparison (Figure 1E), DeepSeek had a higher grade level in two diseases (CKD: 84.61%; COPD: 90.0%), while ChatGPT had a higher understandability percentage in the other two diseases (epilepsy: 81.81%; heart failure: 86.36%).

When the actionability percentages of the educational guide were compared (Figure 1F), it was found that ChatGPT had the same percentage of actionability on all four diseases (50%). DeepSeek had a higher actionability percentage for CKD (75%) and heart failure (87.5%), whereas both ChatGPT and DeepSeek had the same percentage of similarity for COPD and epilepsy (50%).

## Discussion

This cross-sectional study compared responses generated by DeepSeek and ChatGPT for patient education guides on chronic diseases (CKD, COPD, epilepsy, and heart failure). The results (Table 1) revealed no statistically significant differences in word count, average words per sentence, sentence count, average syllables per word, ease score, grade level, DISCERN score, understandability, and actionability between the two AI tools (all p-values >0.05), except for similarity percentage (p-value= 0.049). Notably, the ease score (ChatGPT: 10.33; DeepSeek: 9.03) approached significance (p=0.051) but did not meet the threshold for statistical significance.

AI holds significant potential in simplifying patient education by generating accessible, tailored health information. The American Medical Association advises that health information for patients be written at a reading level of grade 6 or below [22]. In this study, both tools produced content with ease scores compatible with college readability (ChatGPT: 34.35; DeepSeek: 40.38). However, the associated grade levels (ChatGPT: 10.33; DeepSeek: 9.03) far exceeded the recommended benchmarks, indicating overly complex language. This suggests that both AI tool outputs remain unsuitable for average patients. For instance, Zhou M et al. reported similar challenges, where AI-generated texts had higher grade levels than recommended [23]. Notably, DeepSeek’s marginally higher ease score and lower grade level suggest it may prioritize readability slightly better than ChatGPT, though both tools require refinement to meet health literacy standards. However, readability can be improved by additional prompts [24] and paid versions of AI models [25].

Plagiarism in AI-generated medical content remains a concern, as these tools are trained on pre-existing literature, risking inadvertent replication. Plagiarism undermines scientific integrity and may propagate outdated or inaccurate information. In this study, similarity percentages (ChatGPT: 46%; DeepSeek: 32.5%) were relatively high. This finding is consistent with the results by Hassanipour et al., who observed an average similarity rate of 45% in ChatGPT-generated texts using the iThenticate plagiarism detection platform [26]. While DeepSeek produced less similar content, neither tool adhered to the acceptable plagiarism threshold of 15-20% for good academic writing [27].

Both tools produced moderate DISCERN scores (ChatGPT: 47.63; DeepSeek: 46.75), reflecting adequate but incomplete coverage of risks, benefits, and evidence quality. In contrast to the study by Zhou et al. [23], where both ChatGPT 4.o and DeepSeek V3 achieved a DISCERN score above 50, which is considered 'fair,' both models in our study scored below 50, indicating a lower quality of AI-generated content. This suggests that while both ChatGPT 4.0 and DeepSeek V3 represent advanced AI models in generating educational content, their performance was not outstanding, as both achieved scores below 50, suggesting that while the content meets basic standards, it still needs enhancement, especially in delivering more precise, detailed, and thorough information. The main reason for the lower quality scores was the absence of cited references, which aligns with findings from a previous study by Howell [28]. This absence undermines the credibility of AI-generated content, posing a risk if patients rely on information that hasn't been verified. In the absence of citations, patients risk making health decisions based on insufficient or deceptive information, potentially delaying treatment, using ineffective self-care, or underestimating their condition, leading to worse outcomes and increased strain on healthcare systems.

PEMAT assesses understandability and actionability. In our study, we also found high understandability for both tools (ChatGPT: 81.81%; DeepSeek: 80.15%), but actionability scores were notably lower (ChatGPT: 50%; DeepSeek: 65.63%). In comparison, in the study by Singh et al., the understandability scores for AI chatbots ranged from 83.3% to 84.6%, while actionability was uniformly high at 100% across all models [29]. Actionability deficits, such as vague instructions, were the primary reason for the low percentage, highlighting a gap between explaining concepts and guiding concrete steps. For patient education to be effective, materials must balance clarity with actionable advice, an area where AI tools require refinement.

Limitation

Scope and Generalizability limitations

The study was limited in focus because it only looked at two AI tools, both ChatGPT 4.o and DeepSeek V3, omitting other models that may give improved performance. Furthermore, the analysis was limited to English-language content, overlooking key hurdles in making the findings global, including translation accuracy, non-English medical terminology, and cultural adaptation. The study’s focus on chronic diseases also limited its relevance, so that it did not capture the potential of AI in other areas of medicine.

Methodological Limitations

Methodological limitations included the challenge of variability in the outputs of AI systems; it was outside the scope of the study to explore individual variations of AI-generated outputs. AI models have a stochastic component; that is, they could return different outputs in response to the same prompts at different times. However, this study only looked at single instances of each of the models and did not test reproducibility over multiple iterations. Moreover, updates to the algorithms or changes to the prompts could create instability in the consistency of results, which also raises questions about comparability. These methodological shortcomings undermine confidence in the robustness of the findings.

Patient-Centric and Clinical Relevance Gaps

There were no patient perspectives or real-world validation. Readability scores were evaluated, but the difference between simplified language and true patient understanding was not investigated. Above all, the study did not use other metrics that confirm if patients really comprehended or remembered the medical information, nor did it measure if the material is truly usable or if it is just technically simple language.

Temporal and Technical Constraints

AI models are evolving rapidly; newer iterations of the models may correct today’s limitations regarding transparency and clinical utility, meaning the implications of the findings are time sensitive. Lastly, the limited comparison between ChatGPT 4.o and DeepSeek V3 restricted insights into the broader AI landscape, limiting the study’s ability to inform comprehensive best practices for AI integration in healthcare.

## Conclusions

Despite their potential to standardize health information, neither ChatGPT nor DeepSeek met the recommended readability standards for patient education, as both generated materials at a college reading level, exceeding the sixth-grade benchmark advised by the American Medical Association. DeepSeek showed marginally better readability and lower grade levels, but both tools require further refinement to improve accessibility for the average patient.

The reality is that materials generated by AI must not only elucidate concepts in an understandable way, but they also need to instruct patients on how to optimize their health and well-being. So, both ChatGPT and DeepSeek show promise in their ability to generate patient education content, yet much would need to be improved in their readability, originality, quality, and actionability before use as a stand-alone clinical practice resource. Human oversight and iterative refinement remain critical to harnessing AI’s full potential in patient education.
